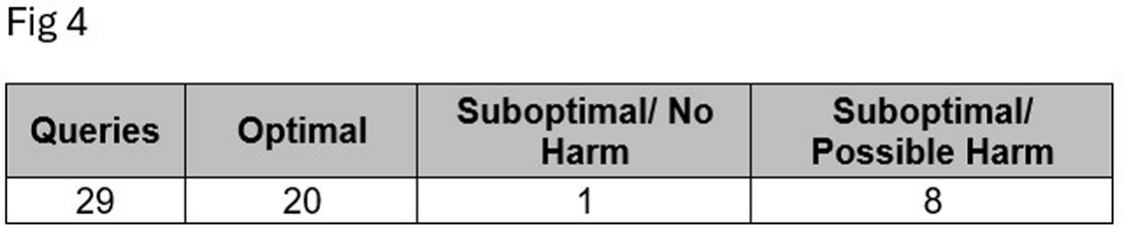# 332 Use of Whole Genome Sequencing to Investigate Potential Sources of Candidozyma auris Transmission in a University Hospital

**DOI:** 10.1017/ash.2026.10737

**Published:** 2026-06-23

**Authors:** Molly Steele, Vera Chu, Palak Patel, Patricia Zuccaro, David Zhang

**Affiliations:** 1 University of Chicago Medicine; 2 University of Chicago; 3 The University of Chicago Medical Center; 4 University of Chicago Medical Center

## Abstract

**Background:** Effective infection prevention and control is integral to the delivery of safe patient care, and with ever increasing demands on their attention, systems to help navigate policy-based guidance can support infection preventionists (IPs) and their healthcare colleagues. Here we present the validation process and results for an internal infection control chatbot. **Methods:** Eleven frequently asked questions were selected to evaluate the performance of ChatUCM, an AI-powered chatbot. Each question was posed five times and response quality was scored by intent recognition, relevance, accuracy, completeness, and consistency. A category pass threshold was set at 75%. IP feedback was incorporated in a ChatUCM update and response quality was scored again. Reference document edits and real-time prompt modifications were made based on post-update scores. A final round of scoring was performed using similar validation metrics. Two IPs scored a subset of five out of the eleven frequently asked questions to assess interrater reliability. Six nursing managers were invited to ask ChatUCM any questions and score its responses by intent recognition, their ability to trust response accuracy, whether their question was fully answered, the achievement of intended goals, and ease of use. 29 questions were submitted. An IP reviewer also scored these ChatUCM responses as optimal, suboptimal/ no harm, and suboptimal/ possible harm. **Results:** The impact of the update on scores was variable. ChatUCM performed well on the same 7 out of 11 questions before and after validation round 1 (Fig. 1). After the final round of retraining, ChatUCM passed all categories with high interrater reliability for our frequently asked questions (Fig. 2). Response quality was less predictable outside of these eleven questions (Fig 3, Fig 4). To ensure its safe use, multiple safety checks were created, including disclaimer language, citation links, education, and a query report to facilitate audits. We encountered multiple surprises while validating ChatUCM. The first update resulted in significant improvements, such as the ability to digest data in table and flow diagram formats. It also saw category scores decrease for six questions, three of which had overall scores drop. ChatUCM struggled with accuracy for questions that required accounting for multiple clinical factors (ex: shingles guidance). **Conclusions:** These findings suggest that ChatUCM could substantially reduce IP workload by accurately answering questions contained in internal SOPs and policies. Additional development will be needed to improve reliability before the tool can function independently without human oversight, particularly when multiple clinical factors must be taken into account.

**Figure:**
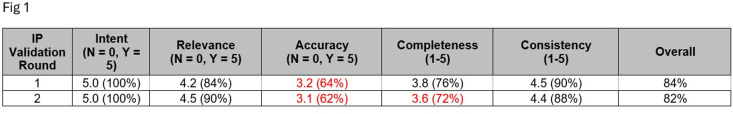

**Figure:**
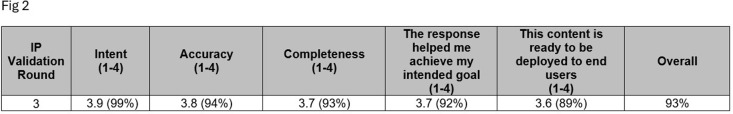

**Figure:**
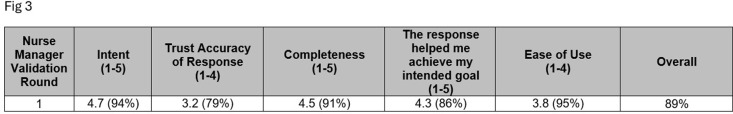

**Figure:**